# Identification of Natural Compounds as Inhibitors of Pyruvate Kinase M2 for Cancer Treatment

**DOI:** 10.3390/molecules27207113

**Published:** 2022-10-21

**Authors:** Iqra Sarfraz, Azhar Rasul, Farhat Jabeen, Tayyaba Sultana, Şevki Adem

**Affiliations:** 1 Department of Zoology, Faculty of Life Sciences, Government College University Faisalabad, Faisalabad 38000, Pakistan; 2 Department of Chemistry, Faculty of Sciences, Cankiri Karatekin University, Cankiri 18100, Turkey

**Keywords:** tumor metabolism, pyruvate kinase M2, phytochemicals, enzymatic assay

## Abstract

The reliance of tumor cells on aerobic glycolysis is one of the emerging hallmarks of cancer. Pyruvate kinase M2 (PKM2), an important enzyme of glycolytic pathway, is highly expressed in a number of cancer cells. Tumor cells heavily depend on PKM2 to fulfill their divergent energetic and biosynthetic requirements, suggesting it as novel drug target for cancer therapies. Based on this context, we performed enzymatic-assay-based screening of the in-house phenolic compounds library for the identification of PKM2 inhibitors. This screening identified silibinin, curcumin, resveratrol, and ellagic acid as potential inhibitors of PKM2 with IC_50_ values of 0.91 µM, 1.12 µM, 3.07 µM, and 4.20 µM respectively. For the determination of Ki constants and the inhibition type of hit compounds, Lineweaver–Burk graphs were plotted. Silibinin and ellagic acid performed the competitive inhibition of PKM2 with Ki constants of 0.61 µM and 5.06 µM, while curcumin and resveratrol were identified as non-competitive inhibitors of PKM2 with Ki constants of 1.20 µM and 7.34 µM. The in silico screening of phenolic compounds against three binding sites of PKM2 provided insight into the binding pattern and functionally important amino residues of PKM2. Further, the evaluation of cytotoxicity via MTT assay demonstrated ellagic acid as potent inhibitor of cancer cell growth (IC_50_ = 20 µM). These results present ellagic acid, silibinin, curcumin, and resveratrol as inhibitors of PKM2 to interrogate metabolic reprogramming in cancer cells. This study has also provided the foundation for further research to validate the potential of identified bioactive entities for PKM2 targeted-cancer therapies.

## 1. Introduction

Cancer has been recorded as the second leading cause of mortality globally, with approximately 9.9 million deaths during 2020 [1]. The tumor heterogeneity, non-selectivity, and toxicity of chemotherapeutic drugs are major hurdles towards successful cancer treatment, which represents an emerging clinical challenge nowadays [2,3]. Therefore, new therapies with enhanced selectivity, increased antitumor potency, and low toxicity are urgently needed.

The recent evidence suggests that targeting the cancer-specific mitochondrial and metabolic remodeling, tumor metabolism, has potential to offer selective treatment for cancer [4]. Cancer cells rewire their metabolic profile by modulating the expression pattern of enzymatic machineries from metabolic pathways (glycolysis, the Krebs cycle, etc.) to fulfil the energy and biosynthetic requirements of highly proliferating cancer cells [5]. Among these rewired metabolic pathways, glycolytic enzymes have emerged as a novel therapeutic target for anti-cancer drug development [6].

The pyruvate kinase is a key regulator of glucose metabolism that encodes for four isoenzymes in cells of mammals [7]. Most of the adult animal tissues have been known to express M1 isoform of pyruvate kinase (PKM1), while the less active isoform of PK (PKM2) is predominantly expressed in embryonic tissues and cancer cells [8]. Tumor cells switch towards PKM2 in order to maintain the pool of glycolytic metabolites to fulfil the biosynthetic requirements of tumor cells [7]. The overexpression of PKM2 has been reported in prostate, breast, lung, hepatocellular, and colorectal cancers. Thus, the inhibition of PKM2 expression in tumor cells could serve as a novel therapeutic approach to halt cancer development and progression [5].

Since PKM2 emerged as a novel target for anti-cancer drugs, it is interesting to discover the inhibitors of PKM2 from natural products (NPs) [5]. Multiple lines of evidence have demonstrated plant-based natural products as a rich database for the identification of metabolic inhibitors [9,10]. Although various inhibitors of PKM2 has been discovered, however, the researches on the discovery of bioactive entities targeting PKM2 are on-going, and the identification of selective, potent, and safer inhibitors could potentially contribute towards the development of effective and safer therapies for cancer patients. Based upon this context, we have performed enzymatic-assay-based screening to evaluate the potential of natural compounds against PKM2. Here, we have provided biochemical, computational, and in vitro evidence that suggests that silibinin, curcumin, resveratrol, and ellagic acid target PKM2 to induce cytotoxicity in cancer cells.

## 2. Results

### 2.1. In Vitro Inhibitory Activity of Phenolic Compounds against PKM2

**Figure 1:**
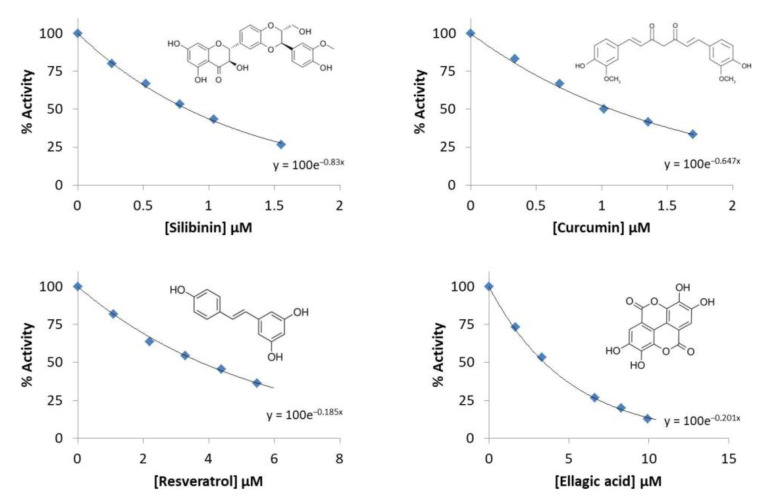
Activity (%) versus phenolics concentration (silibinin, curcumin, resveratrol, and ellagic acid) regression analysis graphs for PKM2.

**Figure 2:**
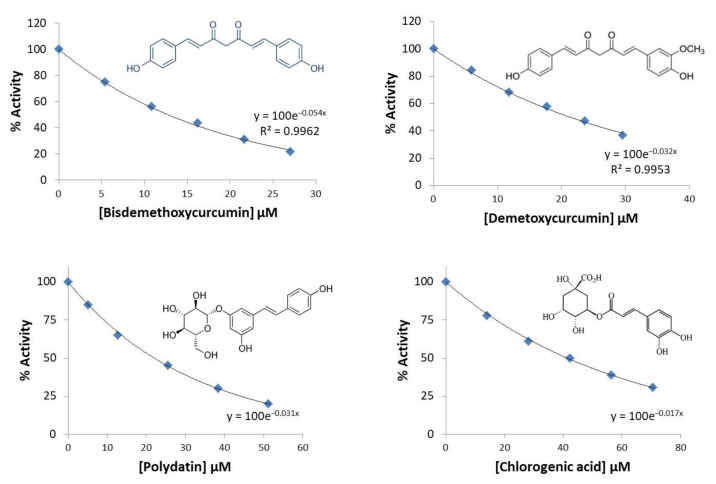
Activity (%) versus phenolics concentration (bisdemethoxycurcumin, demethoxycurcumin, polydatin, and chlorogenic acid) regression analysis plots for PKM2.

In the preliminary screening, the effect of thirty eight phenolic compounds (Table S1) on human PKM2 enzymatic activity was investigated at a single standard dose. To confirm the activity of eight hit compounds, based on their strong inhibitory effects against PKM2, we performed the second potent compounds screen at varying concentrations to calculate their IC_50_ (the concentration of the compound that reduced 50% of the enzymatic activity) values. The IC_50_ values were calculated under comparable enzymatic activity assay by generating dose-response curves (Figure 1 and Figure 2).

The velocity of reaction catalyzed by PKM2 was calculated at saturating concentrations of substrate (PEP) and ADP with six various concentrations of the test compounds.

Results of the study including IC_50_ values are summarized in Table 1.

Among these eight compounds, five compounds exhibited strong inhibitory effects against PKM2 with an IC_50_ value of less than 20 µM. The highest inhibition was expressed by silibinin against PKM2 with an IC_50_ value of 0.91 µM, followed by curcumin, resveratrol, ellagic acid. bisdemethoxycurcumin exhibited moderate PKM2 inhibitory activity with an IC50 value of 16 µM, while three compounds (demethoxycurcumin, polydatin, and chlorogenic acid) were found to possess an IC_50_ value greater than 20 µM.

### 2.2. Determination of Ki Constant Values of PKM2 Inhibitor Phenolic Compounds

**Figure 3:**
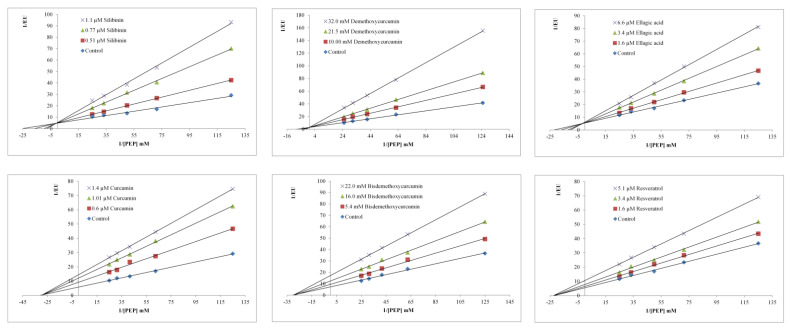
Determination of inhibition type of phenolic compounds by Lineweaver–Burk plots.

For the determination of Ki constants, the Lineweaver–Burk graphs were plotted (1/V versus 1/[S] (Figure 3). The plotted graphs clearly represent that silibinin, ellagic acid, and demethoxycurcumin are competitive inhibitors of PKM2, while curcumin, resveratrol, and bisdemethoxycurcumin performed the non-competitive inhibition of PKM2 enzyme activity. The Ki constants calculated from the Lineweaver–Burk graphs are provided in Table 2.

### 2.3. Determination of PKM2 Binding Affinities of Hit Compounds by Molecular Docking Analysis

**Figure 4:**
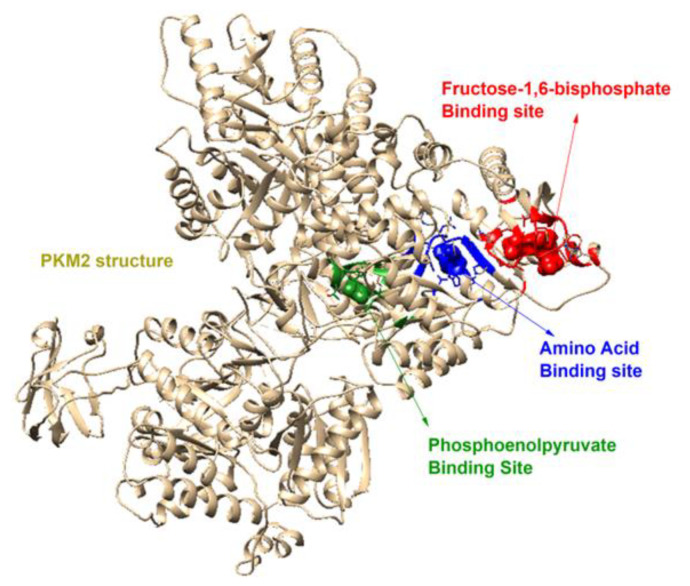
Representation of selected PKM2 docking cavities.

The docking of phenolic compounds was performed against PEP (phosphoenolpyruvate), FBP (fructose-1,6-bisphosphate), and the amino-acid binding sites of PKM2, to understand their binding pattern, with the target protein using the Molegro virtual docker and AutoDock Vina. The selected binding sites of PKM2 are depicted in Figure 4.

The obtained docking scores for each binding site of PKM2 are presented in Table 3.

**Figure 5:**
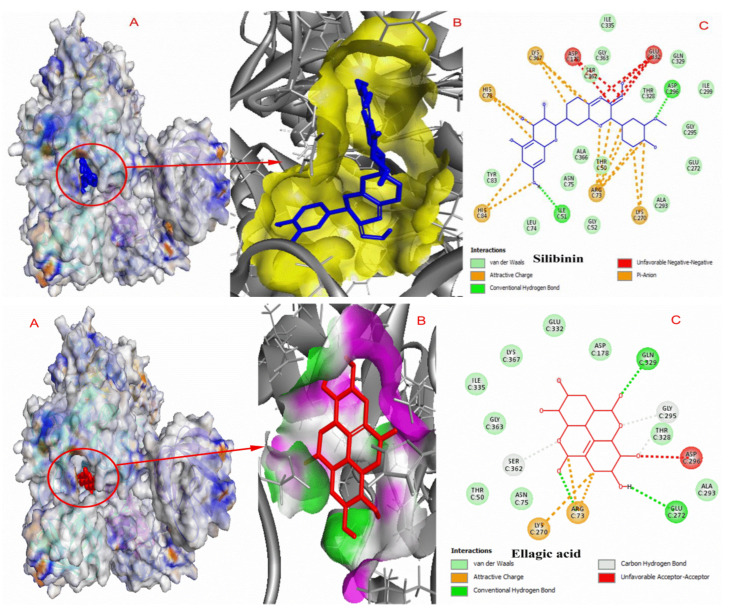
Representation of the PKM2 protein–ligand complexes. (**A**) The binding site of the molecule in the crystal structure of the PKM2; (**B**) molecule at the PKM2 active region; and (**C**) 2D interaction map of the molecule with amino-acid residues of PKM2.

The results of the in silico studies that validated the inhibition mode of compounds obtained by in vitro studies are highlighted in bold. Silibinin and ellagic acid possess good binding energies (kcal/mol) with the PEP binding site of PKM2, indicating that these compounds bind to PEP binding sits and perform the competitive inhibition of PKM2. The interaction modes of silibinin and ellagic acid at the PKM2 binding cavity are shown in Figure 5.

**Figure 6:**
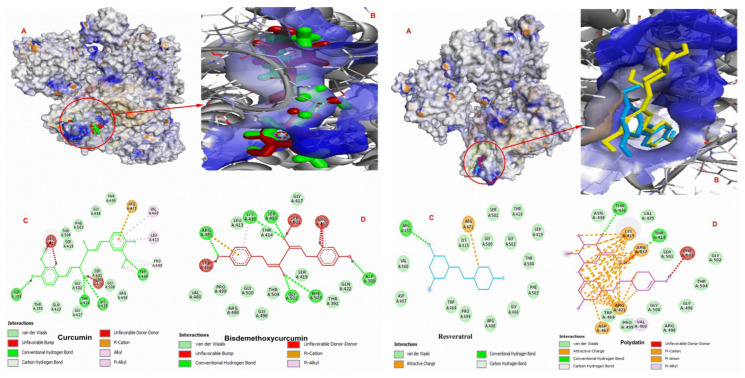
Docking complexes of curcumin, bisdemethoxycurcumin, resveratrol, and polydatin with PKM2. (**A**) The binding site of the ligand in the crystal structure of the PKM2; (**B**) ligand interacting with the active region of PKM2; and (**C**,**D**) amino residues interacting with the compound in the active site of PKM2.

**Figure 7:**
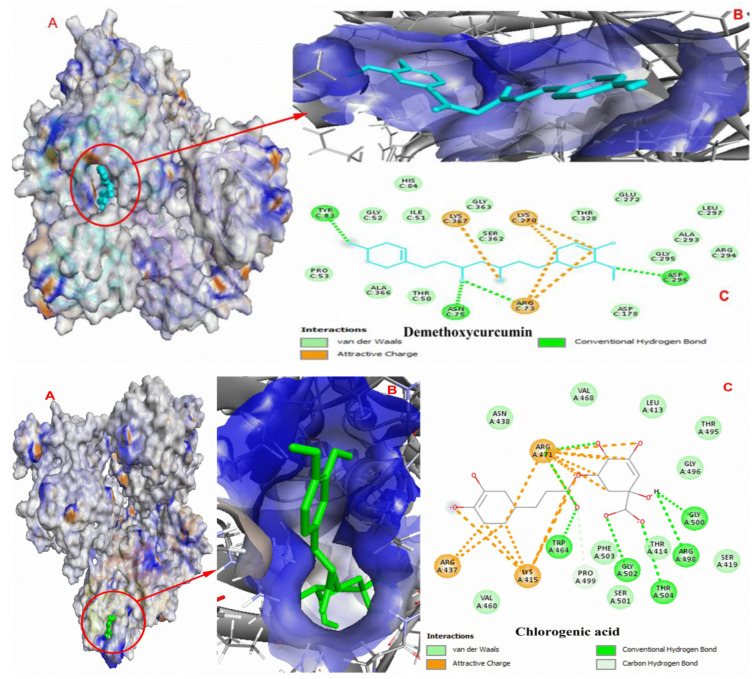
Molecular docking complexes of ligands with PKM2. (**A**) Binding site of ligand in the crystal structure of PKM2, (**B**) ligand interacting with the PKM2 active region, and (**C**) amino residues involved in the protein–ligand interactions.

Docking complexes of curcumin, bisdemethoxycurcumin, resveratrol, polydatin, demethoxycurcumin, and chlorogenic acid are shown in Figure 6 and Figure 7, respectively.

The results of docking studies indicate that these ligands exhibit good binding efficacy with the PKM2 protein by making electrostatic and hydrophobic interactions as well as hydrogen bonds. LYS270, ARG489, and ARG73 were found as common amino acids forming interactions with the docked ligands. Table 4 shows the interaction details between hit compounds and the amino-acid residues of PKM2.

### 2.4. Anti-Cancer Potential of PKM2 Inhibitor Compounds against TNB (Triple-Negative Breast) Cancer Cells

**Figure 8:**
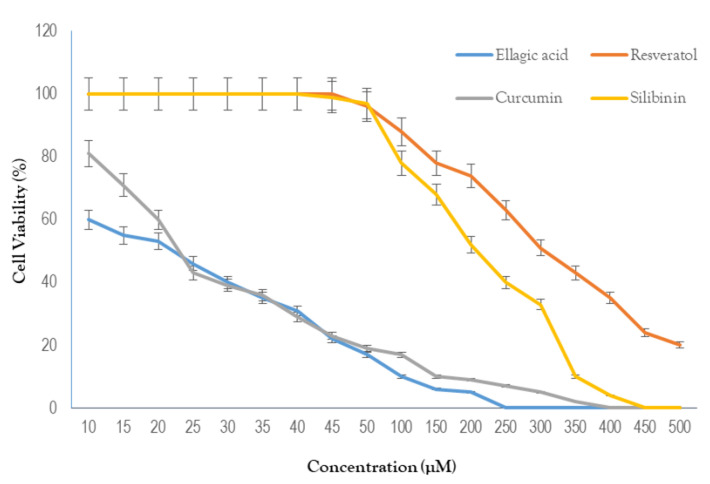
Dose-response curves of hit compounds against MDA-MB231 cells.

The results of our biochemical and in silico studies led us to investigate whether the PKM2 inhibitor compounds possess cytotoxicity towards breast cancer cells. Higher expression of the PKM2 protein has been reported in MDA-MB231 cells [5]. In addition, the MDA-MB231 cell line is preferred as a pre-clinical cancer model due to its highly proliferative nature in vitro as well as in vivo [11]. Thus, MDA-MB231 cells were selected as a model in order to investigate the anti-breast cancer potential of hit compounds. The obtained results clearly indicate that these compounds showed dose-dependent inhibition of the growth of cancerous cells (Figure 8).

The calculated IC_50_ values are represented in Table 5. According to the obtained results, ellagic acid and curcumin were found to be the most potent inhibitors of cancer-cell growth, with IC_50_ values of 20 and 26 µM, respectively.

## 3. Discussion

Aerobic glycolysis is an emerging metabolic signature of cancer cells [12]. The well-established role of PKM2 in tumor glycolysis and TNBC growth indicates that it is a novel drug target for TNBC. In addition, PKM2 inhibition possesses no effects on normal mammary tissues, further supporting its role for cancer treatment [13]. Based on this context, we tried to identify the inhibitors of PKM2 from NPs for the treatment of breast cancer. Here, we have presented biochemical and computational in silico evidence suggesting that silibinin, curcumin, ellagic acid, and resveratrol directly target PKM2 to exert their anti-cancer effects.

Ellagic acid, a polyphenolic compound, is naturally found in pomegranates, berries, and dry fruits. Ellagic acid is endowed with strong anti-cancer properties with inhibitory potential against metastasis and angiogenesis [14]. Although ellagic acid has been already known as anti-cancer agent, this is the first study that demonstrated that ellagic acid acts as tumor-metabolism modulator, binds with PKM2 at the active site, and inhibits its activity in cancer cells. The identification of ellagic acid as a PKM2 inhibitor is a novel finding of this study. Our findings are in line with recently reported data demonstrating the potential of ellagic acid to induce apoptosis in MCF-7 and MDA-MB-231 cancer cells with IC50 values of 23 µM and 27 µM, respectively [15]. A previous toxicity study has also revealed ellagic acid as a safer compound to the concentration of 39 g/kg body weight of test animals with no histopathological signs or treatment-associated clinical alterations [16], further validating its potential for therapeutic applications. 

Silibinin is a nature-derived polyphenolic compound that possesses anti-cancer effects via apoptosis induction, cell-cycle arrest, and inhibiting metastasis [17]. Silibinin is also a major active constituent of Silymarin, which is promising drug used for various ailments [18]. Silibinin also possess synergistic effects in enhancing paclitaxel drug toxicity in gastric cancerous cells [19], supporting the claim that metabolic inhibitors in combination with chemo drugs have the potential to cure cancer. Despite indications that silibinin is an anti-cancer agent, the mechanism of action or molecular target of silibinin still remains to be elucidated. Silibinin has been previously known to decrease the levels of PKM2 mRNA and protein in TNBC cells. Metabolomic analysis illustrated that silibinin inhibited the synthesis of glycolytic ATP, glucose uptake, and NADPH production in TNBC cells. Silibinin-induced reduced glucose utilization and glycolytic flux starved other metabolic pathways fed by glycolysis products and intermediates. Further, the silibinin-induced reprograming of cancer metabolism led to reduced cellular proliferation and stemness with enhanced combinatorial chemotherapeutic (Taxol) outcomes. In addition to PKM2, silibinin also inhibited the activity of HK2 in TNBC cells. All of these findings support the results of this study, suggesting that targeting TNBC metabolism by silibinin has the potential to improve clinical outcomes in TNBC patients [20]. 

Curcumin is an anti-cancer polyphenolic nutraceutical derived from *Curcuma longa* [21]. Curcumin has been known to exert its anticancer effects via the inhibition of the cell cycle, the Warburg effect, and metastasis. Our results are in line with previous studies demonstrating that curcumin inhibits cancer metabolism and suppresses the expression of the PKM2 protein. The overexpression of PKM2 reversed the inhibitory effects of curcumin on tumor glycolysis [22], suggesting that the curcumin-induced inhibition of tumor metabolism was mediated by PKM2. 

Resveratrol is a stilbene-polyphenolic compound that is abundantly found in grapes. A plethora of in vitro, in vivo, and preclinical studies has reported the anticancer properties of resveratrol in various cancer types [23]. Resveratrol has also been reported previously to down-regulate the expression of PKM2 and suppress cancer metabolism, which support the results of this study. Resveratrol has the potential to reduce PKM2 mRNA and protein by two folds in various cancer cell lines (HeLa, MCF-7, and HepG2). Further, the overexpression of PKM2 counteracted the effects of resveratrol, which provides evidence for the PKM2-mediated suppression of tumor glycolysis by resveratrol [24]. 

Although findings of this study revealed ellagic acid, silibinin, resveratrol, and curcumin as tools to interrogate cancer cell metabolic plasticity, it is also imperative to investigate the potential pre-clinical implications of these results.

## 4. Materials and Methods

### 4.1. Chemicals

All of the chemicals and recombinant PKM2 protein were acquired from Sigma Chemical Co. (Saint Louis, MO, USA).

### 4.2. PKM2 Activity Assay

The activity assay used for PKM2 was based on spectrophotometric measurements using a Rayleigh UV-2601 UV/VIS spectrometer set at 340 nm, where we detected the NADH decreasing with lactate dehydrogenase activity. The absorbance value was measured after every 1 min. The total sample volume was 1 mL. Each sample contained 50 nanogram recombinant enzyme, 5 mM MgCl2, Tris pH 7.5 (50 mM), 100 mM KCl, ADP (0.6 mM), 0.5 mM PEP (Phospho-enol pyruvic acid), 180 μM NADH (β-Nicotinamide adenine dinucleotide), 8 units of lactate dehydrogenase (LDH), and 10 μM of FBP (Fructose 1,6-bisphosphate). All of the enzymatic reactions were repeated three times [5].

### 4.3. Inhibition Studies

Compounds were dissolved in DMSO (1 mg/mL) and were diluted ten times with water. These compounds were tested for their enzymatic inhibitory activity at different concentrations. The reaction mixture and inhibitor except PEP were incubated for 10 min. The reaction was initiated by the adding substrate. Enzyme activity without inhibitors was used as the control, and the percent activity caused by them was calculated in proportion to the activity of the control using the corresponding absorbance values. To calculate the IC_50_ values of compounds, % activity versus inhibitory concentration graphs were drawn using Microsoft Excel 2010 [25].

### 4.4. Kinetic Studies

The measurement of PKM2 inhibition was carried out by varying the concentration of PEP in the presence of three various concentrations of phytochemicals. Enzyme velocities were assessed from the change in absorbance per minute up to 5 minutes from the beginning of the reaction. The inhibition types of compounds on the enzymatic activity were determined by Lineweaver–Burk graphs (1/V) (inverse of velocities) versus 1/[S] µm^−1^ (inverse of substrate concentration). The inhibitor constant, Ki, was computed by using the formula slope = Km/Vmax (1 + I/Ki) obtained from these graphs [25].

### 4.5. Molecular Docking

The structure of human PKM2 was obtained from a protein data bank (PDB) (www.rcsb.org (accessed on 31 August 2022)) that had PDB ID 6V74. The target protein structure was prepared for docking using UCSF Chimera 1.10.1 software (1.10.1, San Francisco, CA, USA). Missing residues were repaired and minimized at Molegro Virtual Docker 7.0. PEP, FBP, and amino-acid binding sites were selected as docking regions. The compound’s 3D structures were obtained from PubChem. Ligand docking simulations were employed on all the compounds against the crystal structure of PKM2 using the Molegro Virtual Docker and AutoDock Vina in the UCSF Chimera platform. The grid box centers were adjusted as reference ligands. For validation, the reference ligands were re-docked with different parameters and the docking procedure was continued with the parameters with the RMSD value below 2. The compounds were docked against selected cavities of the PKM2 enzyme, with ten numbers of runs for each docking. The 3D graphics of docking poses were depicted using Discovery Studio 2021 Client.

### 4.6. MTT Assay

The cytotoxicity of phenolic compounds towards cancerous cells was evaluated by MTT assay. Cancer cells were cultured in 96 well plates. The cancer cells were treated at varying concentrations of test drugs for 48 h. After 48 h of the treatment, 10 µL of MTT (5 mg/mL) solution was mixed, and incubation was carried out at 37 °C for 4 h. Subsequently, 150 μL of DMSO was used for dissolving the formazan crystals, and absorbance was obtained at 570 nm at a microplate reader [26]. The absorbance of the control cell and cells with treatment were used to calculate the cytotoxicity of compounds.

I% = (A570 (control) − A570 (treated))/(A570 (control)) × 100

## 5. Conclusions

This study screens plant-derived natural compounds by PKM2 enzymatic activity assay to find out the potent inhibitors of PKM2 that can be utilized as drug leads for PKM2-dependent cancers. Silibinin, curcumin, resveratrol, and ellagic acid were identified as potential inhibitors of PKM2. Molecular docking analysis has provided insights into the binding pattern highlighting the possible binding pockets of silibinin, curcumin, resveratrol, and ellagic acid, and important binding residues of PKM2. Conclusively, this research provides an insight into the molecular target of silibinin, curcumin, resveratrol, and ellagic acids as anti-cancer agents. As PKM2 is a rate-limiting enzyme of glycolysis; therefore, it is recommended to detect the effect of these compounds on the expression of other key proteins of the glycolytic pathway. These results also warrant further detailed therapeutic evaluation of identified inhibitors for PKM2 targeted anti-cancer therapies.

## Figures and Tables

**Table 1:** The results of in vitro studies of some phytochemicals against PKM2.

Compound Name	IC_50_ Values (µM)	Effect
Silibinin	0.91	Inhibition
Curcumin	1.12	Inhibition
Resveratrol	3.07	Inhibition
Ellagic acid	4.20	Inhibition
Bisdemethoxycurcumin	16	Inhibition
Demethoxycurcumin	21.38	Inhibition
Polydatin	25.40	Inhibition
Chlorogenic acid	43.40	Inhibition

**Table 2:** Ki constants for PKM2 calculated from Lineweaver–Burk graphs for different phenolic compounds.

Compound Name	Ki Constants (µM)	The Type of Inhibition for Substrate (FEP)
Silibinin	0.61 ± 0.26	Competitive
Curcumin	1.20 ± 0.40	Non-competitive
Ellagic acid	5.02 ± 0.73	Competitive
Resveratrol	7.34 ± 1.70	Non-competitive
Demethoxycurcumin	14.87 ± 3.03	Competitive
Bisdemethoxycurcumin	18.04 ± 5.80	Non-competitive
Polydatin	-	-
Chlorogenic acid	-	-

**Table 3:** The binding affinities of phytochemicals against PEP, FBP, and amino-acid binding site of PKM2.

Compound Name	Phosphoenolpyruvate (PEP) Binding Site		Fructose-1,6-bisphosphate (FBP) Binding Site		Amino-Acid Binding Site	
	Molegro Virtual-Docking Score	AutoDock Vina kcal/mol	Molegro Virtual-Docking Score	AutoDock Vina kcal/mol	Molegro Virtual-Docking Score	AutoDock Vina kcal/mol
Silibinin	−133.086	**−9.8**	−148.074	−7.3	−111.364	−1.2
Curcumin	−148.904	−8.2	**−162.057**	−7.3	−123.1	−5.4
Resveratrol	−121.483	−7.1	−118.621	**−6.9**	−148.976	−4.6
Ellagic acid	−80.5511	**−8.0**	−94.4214	−5.3	−149.257	−5.8
Demethoxy- curcumin	−149.617	**−7.8**	−160.966	−7.4	−144.438	−5.3
Bisdemethoxy- curcumin	−137.166	−7.6	**−154.467**	−7.2	−88.9558	−5
Polydatin	−131.792	−8.2	−150.452	**−8.1**	−87.9797	−6.4
Chlorogenic acid	−133.36	−7.9	−149.592	**−8.0**	−132.646	−6.1

Bold numbers: in silico scores validating the results of in vitro studies.

**Table 4:** Important PKM2 binding residues interacting with phenolic compounds.

Interactions	Silibinin	Curcumin	Resveratrol	Ellagic Acid	Demethoxy- Curcumin	Bisdemethoxy curcumin	Polydatin	Chlorogenic Acid
Hydrogen bond	ASP296 ILE51	THR432 LYS433 TRP482 THR522 ASP407 SER434	ARG437	ARG73 ARG73 GLN329 GLU272	ARG73 ASN75 TYR83 ASP296	LYS433 SER434 ARG489 GLY520 PHE521 SER519 ASP407	THR436 THR414	TRP464 ARG471 GLY502 THR504 ARG498 GLY500
Carbon hydrogen bond	-	-	ARG437	GLY295 GLY295 SER362	-	-	LYS415	PRO499
Electrostatic	ARG342 LYS270 HIS78	ARG489	ARG471	ARG73 LYS270	ARG73 LYS270 LYS367	-	LYS415 ARG437 ASP467	LYS415 ARG437 ARG471
Hydrophobic	-	PRO517 ARG436 LEU431 VAL486 TRP482	-	-	-	LYS433 ARG436	VAL460	-

**Table 5:** IC_50_ values of hit compounds against breast cancer (MDA-MB231) cells.

Compound Name	IC_50_ Value
Silibinin	208 µM
Curcumin	26 µM
Ellagic acid	20 µM
Resveratrol	306 µM

## Data Availability

Not applicable.

## Supplementary Materials

The following supporting information can be downloaded at: https://www.mdpi.com/article/10.3390/molecules27207113/s1, Table S1: Natural compounds library used for the screening of PKM2 inhibitors.

## Sample Availability

A sample of the compound (ellagic acid) is available from the authors.
