# Supplementary material for: Identification of Natural Compounds as Inhibitors of Pyruvate Kinase M2 for Cancer Treatment
Source: Molecules. 2022 Oct 21;27(20):7113. doi: 10.3390/molecules27207113 (PMC9609560; doi:10.3390/molecules27207113)
Supplement: Supplementary file 1 [file molecules-27-07113-s001.zip › molecules-1918174-supplementary.pdf]

Table S1. Natural compounds library used for the screening of PKM2 inhibitors

**Sr. No.**	**Compound Name**	**Sr. No.**	**Compound Name**
1	Cinnamic acid	20	Sinensetin
2	p-Coumaric acid	21	Scopoletin
3	Gallic acid	22	Curcumol
4	Ferulic acid	23	Arbutin
5	Quercetin	24	Vitexin
6	Syringic acid	25	Phloretin
7	Limonene	26	Rutin
8	Polydatin	27	Hesperidin
9	Ellagic acid	28	Berberine
10	Resveratrol	29	Diosmetin
11	Demethoxycurcumin	30	Apigenin
12	Bisdemethoxycurcumin	31	Physcion
13	Curcumin	32	Hispolon
14	Chlorogenic acid	33	Scutellarein
15	Silibinin	34	Vanillin
16	Mangiferin	35	Tangeretin
17	Juglone	36	Nobiletin
18	Rosmaniric acid	37	Formononetin
19	Cardamonin	38	Tubeimoside-I
